# Comparison of Conservative Interventions for Proximal Hamstring Tendinopathy: A Systematic Review and Recommendations for Rehabilitation

**DOI:** 10.3390/sports11030053

**Published:** 2023-02-24

**Authors:** Pilar Dizon, Michael Jeanfavre, Gretchen Leff, Rachel Norton

**Affiliations:** 1 Stanford Health Care Orthopedic Outpatient Center, Redwood City, CA 94063, USA; 2 Body in Balance PT, Fairfield, CA 94533, USA

**Keywords:** conservative interventions, exercise, proximal hamstring tendinopathy, shockwave therapy, physical therapy

## Abstract

Knowledge of muscular forces and adaptations with hamstring-specific exercises can optimize exercise prescription and tendon remodeling; however, studies investigating the effectiveness of the current conservative management of proximal hamstring tendinopathy (PHT) and outcomes are lacking. The purpose of this review is to provide insights into the efficacy of conservative therapeutic interventions in the management of PHT. In January 2022, databases including PubMed, Web of Science, CINAHL, and Embase were searched for studies assessing the effectiveness of conservative intervention compared with that of a placebo or combination of treatments on functional outcomes and pain. Studies that performed conservative management (exercise therapy and/or physical therapy modalities) in adults 18–65 years were included. Studies that performed surgical interventions or whose subjects had complete hamstring rupture/avulsion greater than a 2 cm displacement were excluded. A total of 13 studies were included: five studies compared exercise interventions, while eight studies investigated a multimodal approach of either shockwave therapy and exercise or a hybrid model incorporating exercise, shockwave therapy, and other modalities, such as ultrasound, trigger point needling, or instrument-assisted soft tissue mobilization. This review supports the notion that the conservative management of PHT may best be optimized through a multimodal approach incorporating a combination of tendon-specific loading at an increased length, lumbopelvic stabilization exercises, and extracorporeal shockwave therapy. With regard to hamstring-specific exercise selection, PHT may be optimally managed by including a progressive loading program at combined angles of the hip flexion at 110 degrees and the knee flexion between 45 and 90 degrees.

## 1. Introduction

Proximal hamstring tendinopathy (PHT) is characterized by localized pain in the region of the ischial tuberosity that is often worsened during running, sitting, and lunging. The hamstring complex suffers the most fatigue and susceptibility to injury during rapid changes that occur from an eccentric to concentric contraction, accounting for an overall injury incidence of 10–55% in recreational and high-performance sports [1,2]. Two types of hamstring injury mechanisms have been described in the literature. Type I injuries, typically seen in sprinters, occur when the hamstring eccentrically contracts during the terminal swing phase, commonly affecting the long head of the biceps femoris (BFlh) proximally [3]. Type II injuries, typically seen in dancers or high-kicking sports, occur during active stretching, as the hip moves into the flexion and knee in extension, commonly affecting the semimembranosus tendon (SMT) and more accurately classified as PHT [3]. In comparison to the quadriceps, which tend to have a low vulnerability to injury, the hamstrings tend to have a greater concentration of type II fibers than the quadriceps, having a greater propensity for fatigue due to this fiber composition [1]. The management of PHT is often challenging due to its multifactorial nature and potential for misdiagnosis due to similarities across differential diagnoses. Current recommendations are drawn from available evidence on pathology and pain in other tendinopathies [4]. Features of tendinopathy include collagen fiber disorganization, angiofibroblastic hyperplasia, and increased mucoid ground substance [5]. The most commonly recommended treatment for PHT is eccentrics, as extrapolated from the management of most tendinopathies [6]. Despite this recommendation, there is a paucity of high-quality evidence to guide clinicians on either exercise prescription (frequency/load intensity/loading angles) or adjunct modalities that may optimize clinical outcomes. The majority of hamstring exercises include knee-dominant hamstring loading, but most runners primarily rely on hip extension to increase their speed [7]. The purpose of the current literature review was to compare the efficacy of conservative interventions for proximal hamstring tendinopathy.

## 2. Materials and Methods

The study question was framed using the PICO format. The PICO question variables, study elements, and respective inclusion and exclusion criteria are shown in Table 1. This literature review was conducted according to The Preferred Reporting Items for Systematic Review and Meta-Analysis (PRISMA) guidelines [8,9]. Between January to June of 2022, the following databases were searched: PubMed, Web of Science, CINAHL, and Embase.

### 2.1. Eligibility Criteria

The inclusion and exclusion criteria for each of the PICO question components are outlined in Table 1.

### 2.2. Search Strategy

The derivation of the search strategy was based upon keywords regarding conservative management and audited by a medical school librarian to ensure the appropriate use of Boolean modifiers, accurate translation of the search strategy across databases, and appropriateness of the search based upon the study’s stated purpose. The search strategies used for each database with the respective results are shown in Supplementary Materials Table S1. The keywords used were variations and derivatives of the following: “proximal hamstring tendinopathy”, “tendon injur*”, and “exercise therapy.” All of the additional materials can be found in the Supplementary Materials file (Tables S1–S18).

### 2.3. Study Selection

**Figure 1:**
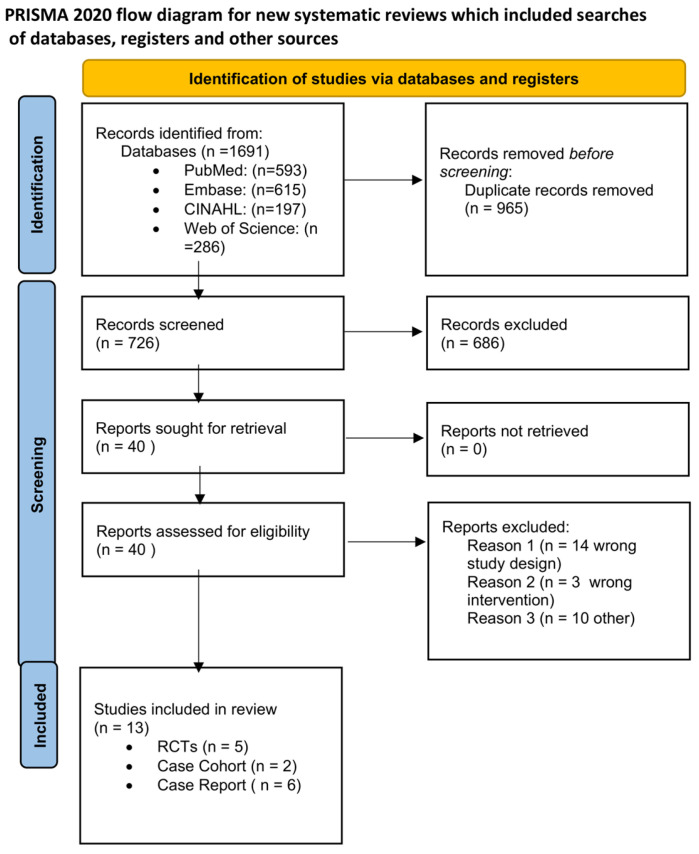
The PRISMA flow diagram.

Search results of the different databases were combined, duplicates were removed, and results were filtered independently according to the specified inclusion and exclusion criteria using a citation manager (Zotero and Covidence) by a pair of independent reviewers (P.D. and R.N.) by title and abstract. Figure 1 outlines the study selection process in a PRISMA flow diagram. Four studies were found by means of a recursive search by the main author. If insufficient information was available through the abstract, full-text analysis was conducted via the above inclusion and exclusion criteria. Consensus was achieved on all publications included in the review without the need to be resolved.

### 2.4. Level of Evidence and Grade of Recommendation

The level of evidence of all included studies was determined according to criteria described by the Oxford Center of Evidence-Based Medicine (CEBM), Oxford United Kingdom (see Supplementary Materials Table S17) [10]. Developed in 1988, the CEBM aimed to make the process of finding appropriate evidence feasible and its results explicit, based on study quality, imprecision, and indirectness, with level I designation being the highest level of evidence [10]. 

The overall Grade of Recommendation for exercise therapy (plus or minus rehabilitative modalities) for the treatment of PHT was determined using The Grading of Recommendations Assessment, Development, and Evaluation (GRADE) Working Group [11]. This hierarchal system was established in the 2000s to develop a common and transparent approach to grading the quality of evidence and strength of recommendations for clinical decisions in health care. Grades of recommendations are in alphabetical order from A–F, with Grade A being the highest level of recommendation (see Supplementary Materials Table S18) [10,12]. 

The inclusion of both of these scales (CEBM Levels of Evidence and the GRADE Working Group criteria) were included by the authors as both scales are endorsed and commonly used in Clinical Practice Guidelines by the American Physical therapy Association (See Supplementary Materials Tables S17 and S18) [12]. 

### 2.5. Data Extraction

Data elements of identified full-text articles were prospectively determined based on the PICO question and the purpose of the current study. Study characteristics with subject demographics, activity level, and study design are detailed in Table 2. The main outcomes assessed in this literature review were pain, as measured by the Visual Analogue Scale (VAS), the Lower Extremity Functional Scale (LEFS), the Victorian Institute of Sport Assessment for Proximal Hamstring Tendinopathy (VISA-H), and return to sport (RTS)/re-injury rate. The LEFS is a validated and reliable tool for assessing functional status in several populations with lower-extremity musculoskeletal conditions [13]. The patient-reported outcome consists of 20 items, with a minimum score of 0 (indicating extreme limitations) and a maximum of 80 (indicating no functional limitations) with a minimal clinically important difference of 9 points [13]. The VISA-H is a patient-reported outcome specially designed to evaluate patients with PHT to assess the severity of symptoms, function, and ability to participate in sports [14]. The outcome consisted of eight questions with a minimum clinically important difference (MCD) of 22 points, with 0 indicating a severe level of disability and the maximum score of 100 points indicating no functional limitations [14]. To compare functional outcome assessments, the LEFS and VISA-H were compared as an overall weighted percent change in disability. The conversion of MCD to a percentage was taken relative to the maximum functional outcome score, with 12.5% improvement being clinically meaningful for the LEFS and 22% being clinically meaningful for the VISA-H. The weighted percentage disability improvement was then calculated by dividing percent improvement by the MCD percentage to account for weight differences. Interventions were assessed as independent categories: strength, lumbopelvic stability, stretching, return to sport, and modality interventions, for further analysis of parameter prescription (frequency, duration of treatment, sets, repetitions, intensity, and intensity).

### 2.6. Risk of Bias Assessment

Once pertinent clinical articles were screened and sorted, methodological quality and risk were independently assessed using the revised Cochrane Risk-of-Bias tool for randomized controlled trials (RoB2), the ROBINS-1 tool Risk-of-Bias in non-randomized studies of interventions, and the Joanna Briggs Institute Critical Appraisal Tool–Checklist for Case Reports. Both ROBINS-I and the Cochrane Rob Tool focus on studies’ internal validity, assessed within specific bias domains by providing signaling questions that flag the potential for bias and should help review authors assess the risk of biased judgment [25]. The Joanna Briggs Institute 2017 Critical Appraisal Checklist (JBI) for Case Reports was used to assess the methodological quality of a study and to determine the extent to which a study has addressed the possibility of bias in its design, conduct, and analysis. The Rating Score is from 1 (lowest) to 10 (highest), with quality scores categorized into three groups: low = 1–4, moderate = 5–7, and high = 8.

## 3. Results

### 3.1. Study Selection and Characteristics

**Figure 2:**
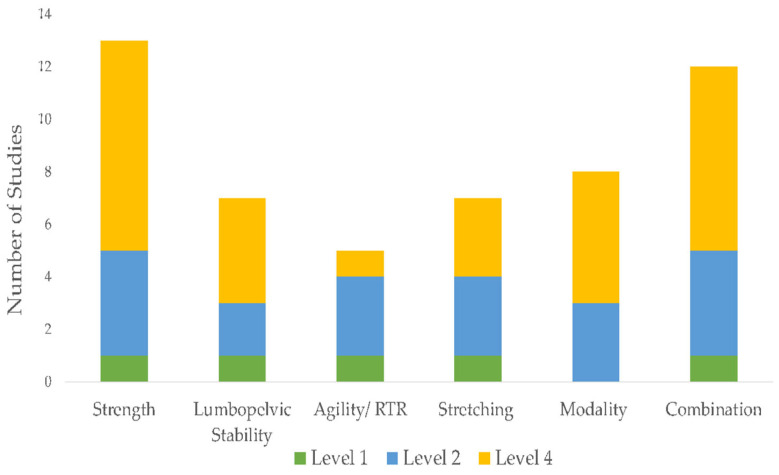
Study interventions by category. Note: Level 1: Evidence obtained from high-quality randomized controlled trials, Level 2: evidence obtained from lesser quality randomized controlled trials, Level 4: case series.

**Figure 3:**
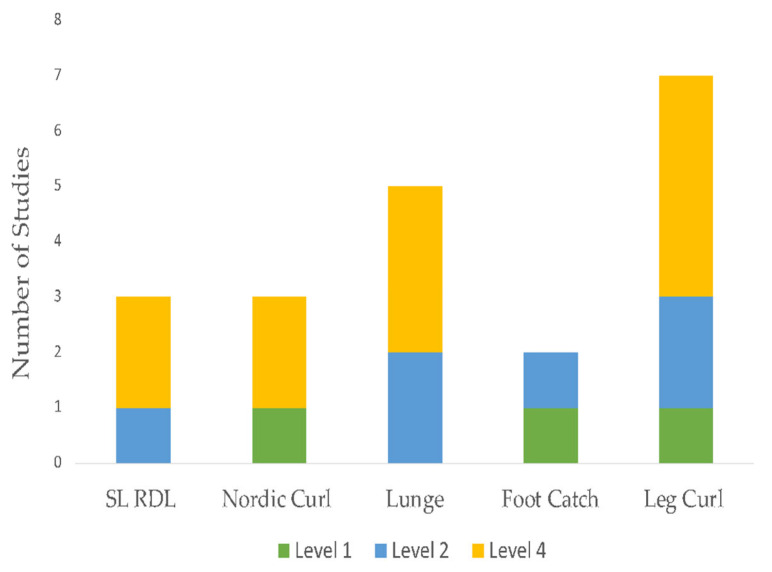
Top five strengthening exercises. Note: Level 1: evidence obtained from high-quality randomized controlled trials, Level 2: evidence obtained from lesser quality randomized controlled trials, Level 4: case series.

**Figure 4:**
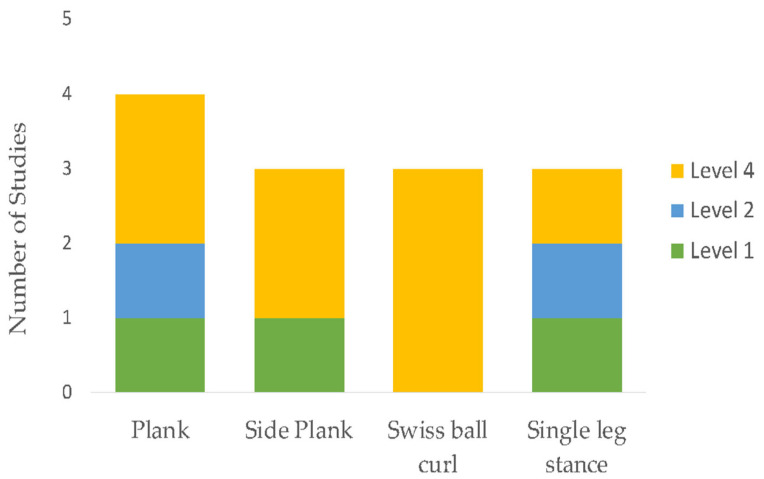
Top four lumbopelvic exercises. Note: Level 1: evidence obtained from high-quality randomized controlled trials, Level 2: evidence obtained from lesser quality randomized controlled trials, Level 4: case series.

**Figure 5:**
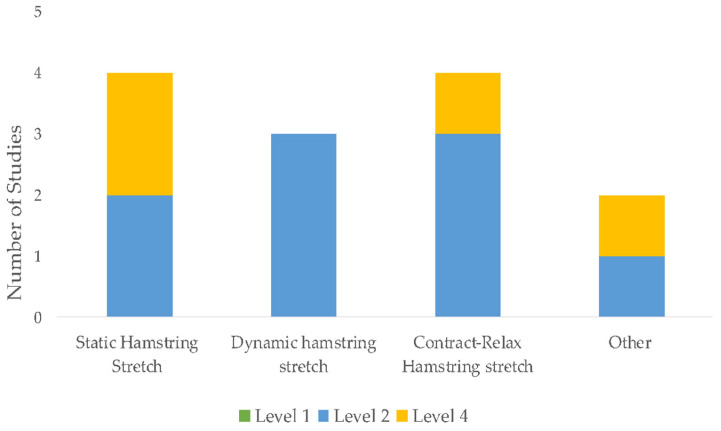
Top four stretching exercises. Note: other denotes specific stretching was not provided, or nonspecific to hamstring muscle group, Level 1: evidence obtained from high-quality randomized controlled trials, Level 2: evidence obtained from lesser quality randomized controlled trials, Level 4: case series.

**Figure 6:**
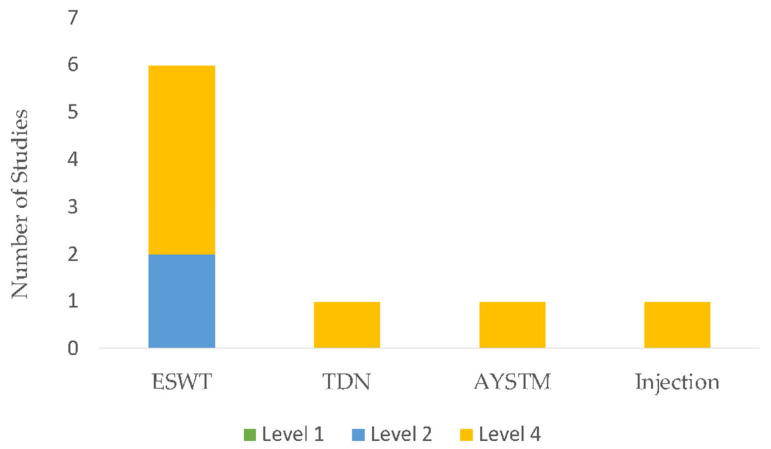
Modalities breakdown by study level. Note: extracorporeal shockwave therapy (ESWT), trigger point dry needling (TDN), instrument-assisted soft tissue mobilization (AYSTM), injection (corticosteroid), Level 1: evidence obtained from high-quality randomized controlled trials, Level 2: evidence obtained from lesser quality randomized controlled trials, Level 4: case series.

**Figure 7:**
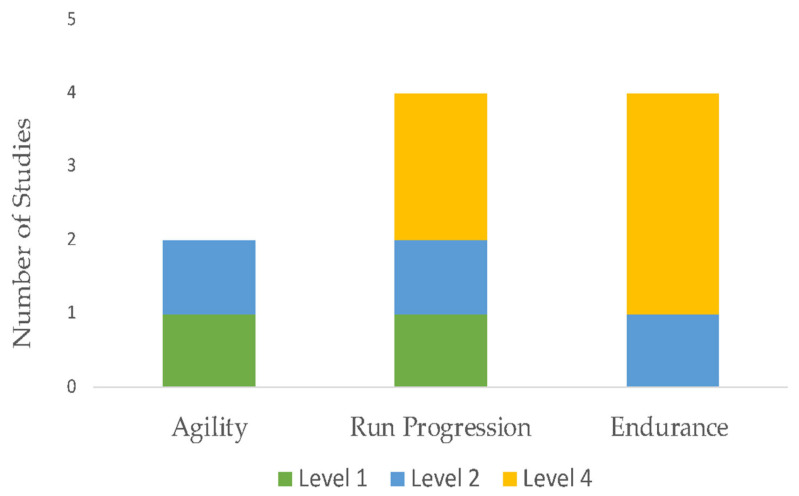
Return to Sport. Note: Level 1: evidence obtained from high-quality randomized controlled trials, Level 2: evidence obtained from lesser quality randomized controlled trials, Level 4: case series.

The initial search identified 1691 unique citations. After filtering the results by tilt, abstract, and full texts via the inclusion and exclusion criteria, 13 articles (5 RCTs, 6 case report studies, and 2 case-cohort studies) were deemed appropriate for final analysis. Five studies compared exercise interventions, and eight studies investigated a multi-modal approach, with the intervention type per study listed in Table 3. Clinical assessments varied between studies ranging from patient-reported outcome scales, such as the Visual Analog Scale (VAS), functional outcomes, such as the Lower Extremity Functional Scale (LEFS), the Victorian Institute of Sport Assessment for Proximal Hamstring Tendinopathy (VISA-H), and return to sport (RTS). Due to the lack of homogeneity of clinical assessments performed, the LEFS and VISA-H were compared as a function of the weighted percent disability relative to published MCDs, to account for weighted differences in change. A summary of outcomes based on the intervention type is listed in Table 4. The intervention composition by study level of evidence is illustrated in Figure 2. The most performed exercises per category are detailed in Figure 3, Figure 4, Figure 5 and Figure 6. The strength parameters used throughout the included studies are listed in Table 5. Return to sport interventions included by studies is listed in Figure 7. Further intervention specificity (strengthening, lumbopelvic stability, stretching, agility/plyometrics, and modalities) is detailed in Supplementary Materials Tables S5–S9. Detailed statistical significance and group differences for individual studies by clinical outcomes are listed in Supplementary Materials Tables S9–S12.

### 3.2. Grade of Recommendations

According to the Grading of Recommendations Assessment, Development and Evaluation (GRADE) Working criteria, interventions that included progressive loading at a minimum RPE of 5 out of 10 at increased muscle length (simultaneous hip flexion and knee extension) and lumbopelvic stabilization performed at least five days a week, with a gradual plyometric (agility/return to run progression), demonstrate a Grade B—Moderate strength of recommendation. This is based on a high-quality randomized controlled trial [15] with findings demonstrating the highest reduction in pain and the fastest recovery to return to sport. Loading regimens should be sustained for a minimum of eight weeks with ESWT performed as an adjunct treatment to a multi-modal approach, rather than a standalone treatment, which demonstrates a Grade C—Weak strength of recommendation. This is based on a single level II study [16] and a preponderance of level IV studies [7,15,16,17,18].

### 3.3. Risk of Bias Assessment

**Figure 8:**
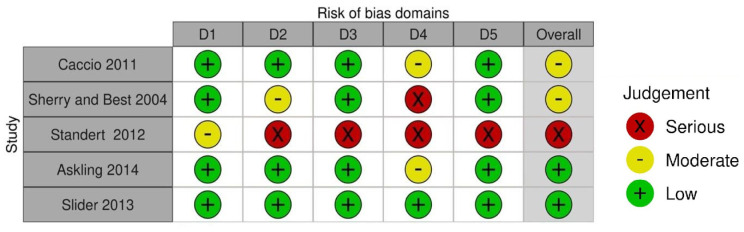
Summary of risk of bias assessment for randomized control trials. Note: Domains: D1: bias arising from randomization process, D2: bias due to deviations from intended intervention, D3: bias due to missing outcome data, D4: bias in measurement of the outcome, D5: bias in selection of the reported result [15,16,19,20,25].

**Figure 9:**
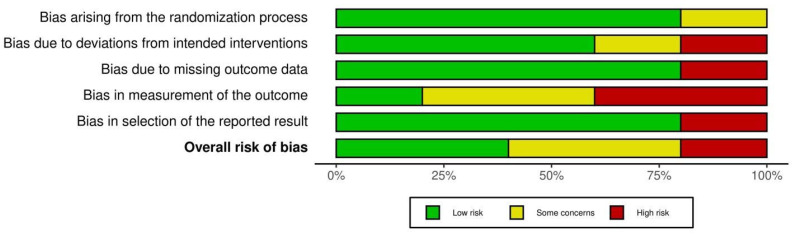
Graphical summary of risk of bias assessment for randomized control trials.

**Figure 10:**
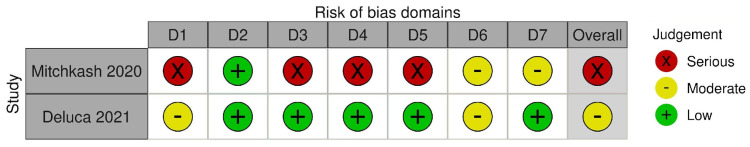
Summary of risk of bias assessment for non-randomized control trials. Note: Domains: D1: bias due to confounding, D2: bias due to selection of participants, D3: bias in classification of interventions, D4: bias due to deviations from intended interventions, D5: bias due to missing data, D6: bias in measurement of outcomes, D7: bias in selection of the reported result [21,24,25].

**Figure 11:**
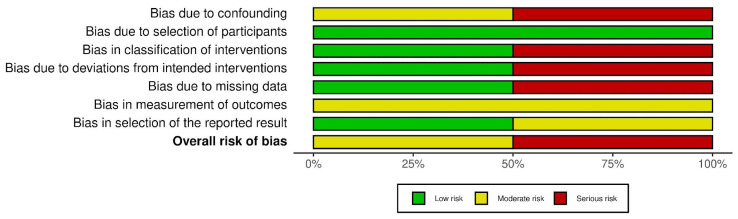
Graphical summary of risk of bias assessment for randomized control trials [26,27].

The RoB assessment results for RCTs are summarized in Figure 8, and a graphical representation of the results is shown in Figure 9. The highest risk of bias was Standert et al. 2012. The lack of blinding in rehabilitation and physical therapy literature is well documented and the RoB assessment results in this review further corroborate this limitation (Armijo-Olivo, S. 2017). However, the majority of RCTs were deemed to have a low risk of bias (see Figure 9). The ROBINS-I assessment results for non-RCTs are summarized in Figure 10, and a graphical representation of the results is shown in Figure 11. The highest risk for bias for the ROBINS-I for non-RCTs was the Mitchkash et al. 2020 study. JBI assessment results are summarized [Table 6], with three studies appraised as high-quality [1,2,3] and three as moderate-quality [4,5,6].

Risk of Bias assessment list is as follows:

1. Were the patient demographic characteristics clearly described?
2. Was the patient’s history clearly described and presented as a timeline?
3. Was the current clinical condition of the patient presentation clearly described?
4. Were diagnostic tests or assessment methods and the results clearly described?
5. Was the intervention(s) or treatment procedure(s) clearly described?
6. Was the post-intervention clinical condition clearly described?
7. Were adverse events (harms) or unanticipated events identified and described?
8. Does the case report provide takeaway lessons?

## 4. Discussion

This literature review aimed to evaluate current conservative interventions and their effectiveness in treating proximal hamstring tendinopathy. To the authors’ knowledge, this is the first literature review evaluating conservative management exclusively. Due to the heterogeneity in outcome reporting, strength parameters, and objective clinical assessments, specific recommendations regarding exercise selection and the contraction type remains uncertain and warrant further investigation with specificity to the proximal hamstring tendon. The type of contractions utilized across studies varied from isometric, concentric, and eccentric, with the vast majority performing a combination of the three. Despite this aforementioned heterogeneity of the studies in this review, the synthesis of outcomes of pain, function, and the RTS/re-injury rate expands on existing knowledge by identifying key considerations with respect to PHT rehabilitation: hip and knee joint angle loading for tendon specificity, load intensity (rather than contraction type), and the importance of lumbopelvic stabilization.

### 4.1. Pain Outcome

Moderate- [17] to high-quality [7,15,18] evidence supports progressive intensity of exercise interventions using a multi-modal approach, which may optimize the reduction in VAS. Of the included studies in this review, the most significant reductions in VAS with the lowest final ratings were reported by Slider et al., 2013 (9-point reduction to 0/10) [15], Cushman et al., 2015 (7-point reduction to 0/10) [7], McCormack et al., 2012 (6 point reduction to 0/10) [18], and Jayasleen et al., 2014 (5 point reduction to 0/10) [17]. Similarities across these studies include increasing progressive intensity with exercise interventions, and all consisted of a multi-modal approach of strengthening, lumbopelvic stability, and endurance training. A differentiating component of the Slider et al., 2013, study, a high-quality RCT, that is consistent with current tendinopathy management, was the use of increasing-effort hamstring isometrics [15]. Isometrics have been substantiated to be beneficial due to the activation of the endogenous opioid system, which has been associated with changes in pain sensitivity, improvements in neural adaptations, and increases in force output. Contrary to the expectation that eccentric training for the management of tendinopathies should yield superior clinical outcomes, Slider et al., 2013, also found a higher reduction in the progressive agility and trunk stabilization (PATS) group (9 points) compared to that in a progressive running and eccentric (PRES) group (5 points) [15]. Recent RCTs have found no superior effect with a high-load magnitude compared to that with a moderate load (55% of 1 RM) on clinical outcomes, tendon structure, and function [28,29] when performed at a slow 3-1-3 concentric and eccentric tempo. Therefore, clinically, it is important to choose the exercise intensity that matches the patient’s tendon load tolerance performed at a slow tempo [4]. Moreover, progressive heavy slow resistance has been shown to achieve greater collagen turnover than sub-maximal eccentric loading [4]. The progressive intensity of exercise interventions in the above-mentioned studies with the highest observed reductions in VAS also underscores that the key to preventing further matrix destruction is through appropriately progressed loads to the tendon to promote remodeling and reduce hypervascularization by reducing the tenocyte expression of vascular endothelial growth factor [30].

### 4.2. Percent Disability as Measured by the LEFS/VISA-H

Moderate- [17] to high-quality [7] evidence supports programs that allow for a maximum of 3/10 pain with combined strength, lumbopelvic stabilization, and progressive endurance components. Relative to established MCDs [13,14], all studies exceeded the MCD percentage; however, two studies that demonstrated the highest weighted percent improvement in disability were the Cushman et al., 2015, study (60% improvement exceeding MCD by 2.72), followed by Jayasleen et al., 2014 (16.2% and 13.7% improvement exceeding MCD by 1.44 and 1.22) [7,17]. Similarities across both studies include a dosage of 3 by 15 performed daily, with a focus on strengthening, lumbopelvic stabilization, and progressive endurance training. Of all included studies in this review, the only two studies that determined load intensity until pain was present with contraction (at maximum 3/10) were the two studies mentioned above. The significant improvement in functional outcomes observed by these two case reports suggests clinicians may be potentially underloading patients, should they encourage only pain-free loading. Further high-quality evidence, however, is necessary to strengthen these claims. Differentiating components across the two studies included baseline characteristics and exercise dosage. Cushman et al., 2015, prescribed an increased dosage of twice a day for twelve weeks, as opposed to once a day for 8 weeks, as in Jayasleen et al., 2014 [6]. This suggests a positive correlation among the volume, duration of treatment, reduction in percent disability, and patient age. Patient activity level and the sport of choice were similar (triathletes); however, the subject in Cushman et al., 2015, was half the age of that in Jayaseelan et al., 2014 (34 years old vs. 69 and 71 years old, respectively). Age may have played a role in the results observed due to an impaired tendon-healing response associated with age due to decreased matrix deposition and decreased stem cell proliferation [31].

### 4.3. Return to Sport and Re-Injury Rate

Two RCTs with a low risk of bias [15] and moderate risk of bias [16] support the incorporation of both eccentric strengthening and lumbopelvic stabilization for a reduced re-injury rate and faster return to sport. The two studies that demonstrated the fastest return to sport were the Sherry and Best et al., 2004 (22 days from the start of rehab), and Slider et al., 2013 (25 days from the start of rehab) [15,16]. Both studies demonstrated statistically significant improvements with a progressive agility and trunk stabilization (PATS) program compared to a stretching and strengthening (STS) control group and the progressive running and eccentric strengthening (PRES) group. Although Sherry and Best et al. demonstrated the fastest return to sport, it is important to note this study demonstrated some concerns for risk of bias as determined by the RoB (Supplementary Materials Table S2), due to lack of individual data for return to sport across participants.

Re-injury rates at one year were lowest in the lengthening protocol group [19] (0% compared to 7% in the conventional protocol group), followed by those in the PATS groups in both Silder et al., 2013, and Sherry and Best, 2004 (6% and 7.7% compared to 70% and 23% re-injury in control groups, respectively). The lengthening protocol focused on mainly loading the hamstrings eccentrically, while the conventional protocol consisted of conventional hamstring exercises with less emphasis on eccentrics [19]. The authors hypothesize that the inclusion of exercises with pelvic motor control and stabilization may have impacted re-injury rates at one year across rehabilitation groups [16]. Although the tissue response and cellular adaptation in tendons are independent of the contraction type, other kinetic chain deficits have the potential to increase hamstring-origin stress concentration [4]. Poor trunk stability and sagittal plane dysfunctions with increased anterior pelvic tilt creating increased hamstring strain are associated with hamstring injury [2]. Two considerations for the reduced re-injury rate in the lengthening group [19] that differ from the other studies were the duration of treatment (16 weeks as opposed to 6 (Slider et al., 2013) and 8 weeks (Sherry & Best, 2004)) and the incorporation of both eccentric strengthening and lumbopelvic stabilization exercise selection.

### 4.4. Joint Angle Loading and Kinematic Considerations with Respect to Hamstring Muscular Activation

The most commonly recommended treatment is exercise-based management; however, there is a lack of quality evidence to guide clinicians with regard to specific exercise selection or dosage. Nordics are among the most popularly prescribed exercise due to their high eccentric demand and association with reducing the rate of hamstring injury by 70% [32]. There have been previous discrepancies in the literature regarding patterns of hamstring activation in hamstring-specific exercises, largely due to the complexities of different neural control strategies in eccentric and concentric phases, but also due to variations depending on both the hip or knee dominance within the structure of a given exercise [33]. Recent EMG studies have investigated hamstring muscular forces and fascicle behavior at varying hip and knee flexion angles, which may better enable clinicians to maximize the effectiveness of their exercise prescription for tendon specificity [34].

Different activation profiles within hamstring-specific exercises exist due to the anatomical and biomechanical distinctions among the four hamstring muscles [33]. During the last 25% of the swing phase, hamstring muscles contract eccentrically at an increased length (hip flexed, knee near full extension), with typical histopathologic findings of PHT revealing the semimembranosus tendon is most commonly affected [2,3,35,36,37]. A recent EMG analysis in 2022 demonstrated that although peak hamstring muscle forces were highest in the Nordic hamstring curl, brief high peak forces may reflect less motor unit recruitment and may not provide an effective stimulus for long-term strength adaptations, with preferential recruitment for the biceps femoris short head and semitendinosus [34]. The roman chair and deadlift exercises may be more effective to promote strength increases in the biceps femoris long head and semimembranosus tendons [34]. Another study found at a 45 degrees hip flexion, “the peak activity of the BFl was found between 15° and 30° of knee flexion whilst the ST and SM worked harder between 90° and 105°” [26]. While isometrics have been supported for analgesia in the reactive tendon, isometrics have also gained considerable traction within the strength and conditioning community in response to Van Hooren and Bosch’s theory that the hamstrings act isometrically, rather than eccentrically, during the late swing phase immediately prior to ground contact during sprinting, while series elastic (tendinous) elements stretch and recoil causing the leg to swing forcefully (Van Hooren 2017). Klakhoven et al., 2020, rebutted this by providing data to debunk these postulations stating “the hamstrings [do not] behave isometrically while sprinting…as neither hip nor knee joint angular velocity’s cross the zero mark on *x*-axis of the velocity time graph at the same time which would signify an isometric point during the running cycle” [38].

Due to the velocity component associated with the Nordic curl, relatively low time under tension, and preferential recruitment of BFsh, a possible inference that can explain the mechanism as to why Nordics carry such benefit in hamstring rehabilitation is perhaps not through proximal tendon remodeling, but rather through an increased fascicle length, which thereby decreases stress–strain at the myotendinous junction. Further, heavy and slow loading rates at an approximately 100 degrees hip flexion and 45–90 degree knee flexion may best target the semimembranosus tendon for necessary remodeling. Loading at these specific joint angles, which tends to mimic the mechanism of injury angle associated with running, may induce greater efficacy of intervention through the principle of specificity. Example exercises that load in these specified ranges of motion are standing foot catches [15,16], eccentric treadmill training [7], stool scoot [18], and reverse dumbbell lunges [17], all utilized by the studies that demonstrated the highest reduction in pain [7,15,17,18] and fastest return to sport outcomes [16].

### 4.5. Comparison to Prior Review

Currently, there is only one other review identified with similar scope, aimed at examining interventions and functional outcomes for patients with PHT [39]. Nasser et al.’s review discussed surgical versus non-surgical interventions, which focused on PRP (n = 4), autologous injection (n = 1), corticosteroid injection (n = 2), shockwave (n = 1), and multimodal intervention and PRP (n = 1), with little emphasis placed on loading regimens described in the literature. From a non-surgical perspective, the exclusion of management via exercise loading regimens renders Nasser et al.’s review heavily biased towards interventions performed by physicians, while in contrast, the scope of this review aimed [aims] to provide recommendations for interventions under the scope of the practice of rehabilitative specialists (physiotherapists, strength and conditioning coaches, and athletic trainers). This further underscores Nasser et al.’s own self-identified limitation “interventions such as load management, heavy slow, strength training, platelet-rich plasma vs. placebo and shockwave vs. sham shockwave should be avenues for future research” [39]. From a rehabilitative perspective, Nasser’s paper provides little insight to guide rehab clinicians on exercise considerations, aside from avoiding high levels of compression early on in rehab [39]. The treatment design and prescription (plus or minus other adjunct modalities) are vital components that affect return to sport success and timelines; however, there are currently no data to recommend the superiority of specific loading regimens over another. Our review investigated potential differences that impacted effectiveness across different loading programs. The sub-analyses performed provides further guidance with respect to exercise selection, loading angle specificity given the biarticular nature of the hamstrings, and considerations for exercise program design (incorporation of lumbopelvic stabilization, tendon-specific loading, and progressive plyometrics) to maximize rehab functional outcomes. Though the current review was unable to perform a meta-analysis due to the study design and intervention heterogeneity, the level of current evidence was summarized with a GRADE of recommendation, distinctly unique in its conclusion from the prior review [39].

### 4.6. Limitations with Included Studies

Due to the variability in outcome measures used, heterogeneity across exercise selection and prescription, specificity with load intensity, and loading rate reporting, it is difficult to make a strong recommendation for one mode of intervention over another, but further highlights the need for further high-quality RCTs investigating these parameters for the management of PHT. One single RCT [20] concluded that shockwave therapy was more effective than the multi-modal intervention; however, there were several threats to internal validity, such as increased time allocated to the intervention versus control group, lack of documented adherence to conventional exercise, insufficient exercise duration/intensity as compared to traditional management of tendinopathies (at least 12 weeks of load intensity at least >55% of 1 RM [27,28] compared to 30% 1 RM for 3 weeks) in the study of Caccio et al., 2011. Critical limitations in current studies focusing on shockwave therapy with respect to conservative exercise therapy include load intensity, duration, and exercise selection, which may have biased results towards this passive modality, although outcomes show clinical benefit as an adjunct in combination with multimodal exercise therapy, not as a sole intervention [20,21].

Other variables that warrant further investigation that may have affected clinical outcomes include load intensity specificity and tempo with exercise selection. Of the 13 studies, only two studies were specific in their load intensities either by RPE or by the percentage of 1RM [6,20], with six studies lacking specificity of either “moderate/high load” or “progressive” [7,15,16,17,22,35], and five studies lacking the reporting of exercise intensity in general [5,18,21,23,24]. Furthermore, of the 13 studies, only two [6,7] commented on loading rates associated with exercise, making it difficult to further analyze differences observed in clinical outcomes across studies.

## 5. Conclusions

This systematic review found few high-quality randomized controlled trials and a predominant number of case reports that support recommendations of progressive loading at a minimum RPE of 5 out of 10 at increased muscle length (~100 deg flexion and knee flexion from 90–45 degrees) (Grade B), lumbopelvic stabilization performed at least five days a week (Grade B), for a minimum of eight weeks (Grade C). Based on current evidence, a gradual plyometric progression (Grade C) should guide clinical practice in most clinical situations. Based on a lack of high-quality evidence, ESWT should be used as an adjunct treatment to a multi-modal approach, rather than a standalone treatment (Grade C). Although universal sport demands exist, on an individual level, several intrinsic and extrinsic factors (specific hamstring muscle involvement, kinetic chain deficiencies, limb strength/endurance asymmetries, range of motion limitations) further necessitate a comprehensive, multi-modal and individualized approach for PHT management.

## Figures and Tables

**Table 1:** PICO question and study design inclusion and exclusion criteria.

Question Component	Inclusion Criteria	Exclusion Criteria
Population	Active adults (18–65-years-old) No evidence of tear	Pediatrics (0–18-years-old) Adults with identified hamstring rupture/avulsion (>2 cm displacement)
Intervention	Conservative management (exercise therapy, therapeutic modalities, such as shockwave therapy, ultrasound, instrument-assisted mobilization) under the scope of practice of physical therapists, athletic trainers, coaches, personal trainers, and other rehabilitation specialists	Surgical interventions Injections (plasma rich, autologous)
Comparison	Non-exercise therapy	
Outcome	Visual Analogue Pain Scale (VAS 0–10) Lower Extremity Functional Scale (LEFS) Victorian Institute of Sport Assessment (VISA-H) Return to Sport (RTS)	
Study Design	Randomized controlled trials (RCTs) Case reports Case cohorts	

**Table 2:** Characteristics of included studies.

Study Type/Level of Evidence	Author	Control Group	Intervention Group	Diagnostic Criteria	Age	Gender	Activity Level	Follow-Up Time
Case report//Level IV	Reilly et al. [5]	NA	1	Clinical exam	25	F:2	Ultramarathoner	6 weeks; 10 month
Case report//Level IV	Kreuger et al. [6]	NA	1	Med Dx as assessed via sports physician	31	M	Power lifter	12 week–12 month follow-up
Case report//Level IV	Cushman et al. [7]	NA	1	Ultrasound	34	M	Triathlete (14 local triathlons for 14 years)	12 week
Randomized, double-blind, parallel-group clinical trial//Level I b	Silder et al. [15]	16 PATS	13 PRES	MRI	16–43	6 F 23 M	Runners	MRI and physical examinations conducted after completion of rehabilitation and up to 6 month following return to sport
Prospective randomized comparison//Level II	Sherry & Best et al. [16]	11 PATS	13 STS	Clinical exam	14–49	8 F: PATS M: 18, STST: F: 4. M: 18	Active in sports	4 weeks
Case study//Level IV	Jayasleen et al. [17]	NA	2	Med Dx as assessed via ortho surgeon	71; 69	M	(A) Active running 40–48 km, biking 80 km per week—discontinued due to pain (B) Triathlete	8/10 week = discharge
Case report//Level IV	McCormack et al. [18]	NA	1	Med dx	41	M	Recreational runner	12 week
Prospective randomized comparison//Level II	Askling et al. [19]	28 L protocol	28 C protocol	MRI	15–29	32 F 68 M	Swedish track and field athletes	1 year for re-injuries Number of days to return to full training
RCT/Level II	Cacchio et al. [20]	20 TCM	20 ESWT	MRI T1 and T2 imaging	23	M: 27 F: 13	Professional athletes	pts evaluated before tx, at week 1, 3, 6, and 12 months after end of use
Case cohort//Level IV	Mitchkash et al. [21]	NA	32	Chart reviewed for “running related injury” that was interfered with normal training and ability to compete >7 days	39	M: 10 F: 22	Runners	8 weeks from initiation tx
Case report//Level IV	Fredericson et al. [22]	NA	1	MRI	32	F	Olympic athlete	6 month
RCT//Level II	Standert et al. [23]	Rest and to take 600-mg ibuprofen twice daily for the first week. 2 weeks PT: modalities + strengthening	n = 20 ESWT	MRI	not stated	27 M 13 F	NR	12 month
Case cohort//Level IV	Yun et al. [24]	40 RSWT	23 CSWT	MRI	42	F: 41 M: 22	Runner	2 years

Abbreviations: randomized controlled trial (RCT), traditional conservative management (TCM) [NSAIDS, physiotherapy (2 weeks), exercise program for hamstring muscles (3 weeks)], progressive agility and trunk stabilization (PATS), stretching and strengthening (STS), progressive running and eccentric strengthening (PRES), lengthening exercises (L-protocol), conventional exercises (C-Protocol), radial shockwave therapy (R-SWT), combined shockwave therapy (C-SWT), treatment (tx).

**Table 3:** Study interventions summary.

Intervention (s)	Study	Strengthening	Agility/Plyometrics/Endurance	Lumbopelvic Stability	Stretching	Other Modalities (Shockwave, Needling, etc.)
**Exercise**	Kreuger et al. [6]	✅				
	Cushman et al. [7]	✅	✅	✅	✅	
	Silder et al. [15]	✅	✅	✅		
	Sherry & Best et al. [16]	✅	✅	✅	✅	
	Askling et al. [19]	✅	✅	✅	✅	
**Exercise + Modality**	Reilly et al. [5]	✅	✅			✅
	Jayasleen et al. [17]	✅		✅		✅
	McCormack et al. [18]	✅			✅	✅
	Cacchio et al. [20]	✅				✅
	Mitchkash et al. [21]	✅				✅
	Fredericson et al. [22]	✅	✅	✅	✅	✅
	Standert et al. [23]	✅			✅	✅
	Yun et al. [24]	✅		✅	✅	✅

**Table 4:** Outcomes summary table.

Intervention (s)	Study	Reduction of VAS	Final VAS	Activity-Related Pain	MCD %	Weighted % Disability Improvement	Return to Sport	Reinjury rate (Percentage %)
**Exercise**	Kreuger et al. [6]	−6	2	Initial: unable to sit >30 min 84 d: 2/10 with sitting >30 min 360 d: 2/10 with sitting >60 min	NR	NR	NR	NR
	Cushman et al. [7] ^ẞ^	−7	0	28 d: pain with sitting present 56 d: pain-free sitting	22%	272%	NR	NR
	Sherry & Best et al. [16]	NR	NR	NR	NR	NR	PATS: 22.2 d STST: 37.4 d	STS: 70% PATS: 7.7%
	Askling et al. [19]	NR	NR	NR	NR	NR	L protocol: median: 62 days C protocol: median: 120 days	L protocol: 0 C protocol: 7%
	Silder et al. [15]	PATS: −9 PRES: −5	0	NR	NR	NR	PATS: 25.2 d PRES: 28.8 d	PRES: 23% PATS: 6%
	Jayasleen et al. [17] ^ɑ^	−5	0	NR	11.2%	144%, 122%	NR	NR
**Exercise + ** **Modality**	Mitchkash et al. [21] ^ẞ^	NR	NR	NR	22%	118%	NR	NR
	McCormack et al. [18] ^ɑ^	−6	0	56 d: 2.5 mile pain-free 84 d: 1 mile jog without pain 112 d: 2.5 mi run without pain	11.2%	111%	NR	NR
	Cacchio et al. [20]	SWT: −4.1 TCT: 0.1	SWT: 2.1 TCT: 6.8	SWT: pain not present during activity but resolves within <48 h TCT: pain present during all activities and with ADLs	NR	NR	SWT: 80% in 63 d TCT: none	NR
	Reilly et al. [5]	NR	0	NR	NR	NR	300 d	NR
	Standert et al. [23]	SWT: −5 TCT: −0.2	SWT: 1.8 TCT: 5.5	SWT: pain not present during activity but resolves within <48 h TCT: pain present during all activities and with ADLs	NR	NR	TCT: none SWT: 80% in 63 d	NR
	Fredericson et al. [22]	NR	0	NR	NR	NR	180 d	NR
	Yun et al. [24] ^ẞ^	NR	NR	NR	22%	102%	NR	NR

VAS: Visual Analog Scale (0–10); MCD (minimal clinical difference: 3 pt reduction); % Disability: percent disability as measured by LEFS; MCD: 9 pts or 12% reduction; % Disabilityẞ: percent disability as measured by VISA-H; MCD: 22 pts or 22% reduction; RTS/A: Return to Sport/Activity; SWT: shockwave therapy; TCT: traditional conventional therapy (control group); ADLs: activities of daily living; d: days; PRES: progressive resistive eccentrics and stretching; PATS (progressive agility and trunk stabilization), STS (strengthening and stretching group); L protocol (lengthening protocol), C protocol (conventional training program); MCD % (minimal clinical difference for outcome measure, used either LEFS or VISA-H as a percentage).

**Table 5:** Strength parameter statistics.

	Sets	Reps	Load	Frequency/Week	Duration of Tx
Average	3	11	63% RM	5	8
Range	1–4	6–20	30–90%	2–7	6–24
Standard deviation	0.74	4.2	15.8	2	5.5

**Table 6:** Summary of risk of bias assessment for case reports (JBI).

Study	1	2	3	4	5	6	7	8	Total	Quality
Reilly et al. [5]	Y	Y	Y	N	Y	Y	N	Y	6	Moderate
Krueger et al. [6]	Y	Y	Y	Y	Y	Y	Y	Y	8	High
Cushman et al. [7]	Y	Y	Y	Y	Y	Y	Y	Y	8	High
Jayasleen et al. [17]	Y	N	Y	Y	Y	Y	Y	Y	7	Moderate
McCormack et al. [18]	Y	Y	Y	Y	Y	Y	Y	Y	8	High
Fredericson et al. [22]	Y	Y	Y	Y	Y	Y	N	Y	7	Moderate

## Data Availability

Not applicable.

## Supplementary Materials

The following supporting information can be downloaded at: https://www.mdpi.com/article/10.3390/sports11030053/s1, Table S1: Search Strategies; Table S2: Tool to Assess Risk of Bias (RoB) Assessment of Randomized Control Trials; Table S3: Tool to Assess Risk of Bias (ROBIN-I) in Cohort Studies; Table S4: Tool to Assess Risk of Bias (JBI) in Case Reports; Table S5: Strengthening Interventions; Table S6: Lumbopelvic Stabilization Interventions; Table S7: Stretching Interventions; Table S8: Endurance/ Return To Run Progression; Table S9: Modality Intervention; Table S10: Pain Outcomes; Table S11: VISA-H Outcome; Table S12: Function Outcomes; Table S13: Modality Intervention; Table S14: Pain Outcomes; Table S15: VISA-H Outcome; Table S16: Function Outcomes; Table S17: Oxford Center for Evidenced-Based Medicine 2011 Level of Evidence; Table S18: Grades of Recommendation.
