# Supplementary material for: Comparison of Conservative Interventions for Proximal Hamstring Tendinopathy: A Systematic Review and Recommendations for Rehabilitation
Source: Sports (Basel). 2023 Feb 24;11(3):53. doi: 10.3390/sports11030053 (PMC10053564; doi:10.3390/sports11030053)
Supplement: Supplementary file 1 [file sports-11-00053-s001.zip › sports-2108496-supplementary.pdf]

Supplementary Table S1: *Search Strategies*

Search Terms	Number of studies
**Pubmed**	
**“Proximal hamstring” OR ((“hamstring tendons”[Mesh] OR “hamstring muscles”[mesh] OR hamstring[tw]) AND (tendinopathy[Mesh] OR “tendon injuries”[Mesh] OR “soft tissue injuries”[Mesh] OR injuries[sh] OR injur*[tiab]) AND proximal[tw])**	450
**“Proximal hamstring” OR ((“hamstring tendons”[Mesh] OR “hamstring muscles”[mesh] OR hamstring[tw]) AND (tendinopathy[Mesh] OR “tendon injuries”[Mesh] OR “soft tissue injuries”[Mesh] OR injuries[sh] OR injur*[tiab]) AND proximal[tw]) AND (“Physical Therapy Modalities”[MH] OR “Physical Therapy”[TIAB] OR Rehabilitation[MH] OR Rehabilitation[TIAB] OR Physiotherapy[TIAB] OR "Conservative"[TIAB] OR "Non-operative"[TIAB] OR "Non-surgical"[TIAB])**	143
**Embase**	
**“Proximal hamstring” OR ((“hamstring tendon”/exp OR “hamstring muscle”/exp OR hamstring:ti,ab,kw) AND (tendinitis/exp OR tendinopathy:ti,ab,kw OR ‘tendon disease’/exp OR “soft tissue injury”/exp OR injury:ti,ab OR injuries:ti,ab) AND proximal:ti,ab,kw)**	615
**CINAHL**	
**(“Proximal hamstring” OR (proximal N3 hamstring N3 (muscle OR tendon) N3 (injury OR injuries)) OR (proximal N2 hamstring N3 (tendinitis OR tendinopathy)))**	197
**Web of Science**	
**(“Proximal hamstring” OR (proximal NEAR/3 hamstring NEAR/3 (muscle OR tendon) NEAR/3 (injury OR injuries)) OR (proximal NEAR/3 hamstring NEAR/3 (tendinitis OR tendinopathy OR injury OR injuries)))**	286
**total**	1691

Supplementary Table S2: *Tool to Assess Risk of Bias (RoB) Assessment of Randomized Control Trials*

Study	D1	D2	D3	D4	D5	Overall
Caccio 2011	Low	Low	Low	Low	Low	Low
Sherry and Best 2004	Low	Some concerns	Low	High	Low	Some concerns
Standert 2012	Some concerns	High	High	High	High	High
Askling 2014	Low	Low	Low	Some concerns	Low	Low
Slider 2013	Low	Low	Low	Low	Low	Low

Supplementary Table S3: *Tool to Assess Risk of Bias (ROBIN-I) in Cohort Studies*

Study	D1	D2	D3	D4	D5	D6	D7	Overall
Mitchkash 2020	Serious	Low	Serious	Serious	Serious	Moderate	Moderate	Serious
Deluca 2021	Moderate	Low	Low	Low	Low	Moderate	Low	Moderate

Supplementary Table S4: *Tool to Assess Risk of Bias (JBI) in Case Reports*

Study	1	2	3	4	5	6	7	8	Total	Quality
Krueger 2020	Y	Y	Y	Y	Y	Y	Y	Y	8	High
Jayasleen 2014	Y	N	Y	Y	Y	Y	Y	Y	7	Moderate
Cushman 2015	Y	Y	Y	Y	Y	Y	Y	Y	8	High
McCormack 2012	Y	Y	Y	Y	Y	Y	Y	Y	8	High
Reilly 2018	Y	Y	Y	N	Y	Y	N	Y	6	Moderate
Fredericson 2005	Y	Y	Y	Y	Y	Y	N	Y	7	Moderate

1. Were the patient demographic characteristics clearly described?
2. Was the patient's history clearly described and presented as a timeline?
3. Was the current clinical condition of the patient presentation clearly described?
4. Were diagnostic tests or assessment methods and the results clearly described?
5. Was the intervention(s) or treatment procedure(s) clearly described?
6. Was the post intervention clinical condition clearly described?
7. Were adverse events (harms) or unanticipated events identified and described?
8. Does the case report provide takeaway lessons?

Supplementary Table S5: *Strengthening Interventions*

STRENGTHENING INTERVENTIONS								
**Author**	**Exercise**	**Sets**	**Repetitions**	**Load**	**Tempo**	**Contraction Type**	**Frequency / week**	**Length of Tx (weeks)**
Kreuger et al., (2020)	low bar back squats<br>sumo dead lifts<br>romanian deadlifts<br>trap bar deadlifts<br>goodmornings<br>loaded barbell hip thrusts<br>Single leg Romanian Deadlift<br>single leg hamstring curl<br>reverse dumbbell lunges	3	6-15	RPE 7-9	3-1-3	concentric/ eccentric	3	12
Jayasleen et al.,(2014)	leg curl machine<br>single leg Romanian deadlift<br>standing hip hikes<br>supine bridge walk out<br>sidelying hip abduction<br>single leg windmills<br>lunges<br>nordic curls	3	15	until pain was present with contraction	NR	eccentric	7	10
Mitchkash et al., (2020)	NR	NR	NR	NR	NR	NR	as tolerated	NR
Cushman et al., (2015)	Phase 1 (four weeks): seated hamstring curl, supine hamstring curl<br>Phase 2: instructed to face backward on treadmill while holding on rails at 0.5 mph, with hip and knee extended individual asked to resist motion of forward belt	3	44910	5 seconds	2x/ day	concentric/ eccentric	7*	12
McCormack et al., (2012)	hamstring curls prone<br>resisted hip extension<br>seated hamstring curl 0 to 90 deg<br>OKC quad stregthening<br>stool scoot hamstring<br>unilateral bridge<br>good mornings<br>nordic<br>lunges<br>single leg squat	2-3	10	NR	NR	eccentric	7*	8
Cacchio et al., (2011)	prone leg curls<br>standing leg curls<br>stannding hip flexion<br>standing hip extension<br>dead lift<br>half squat<br>counter movement jump	4<br>3<br>3<br>4<br>3<br>4<br>3	6<br>10<br>10<br>6<br>10<br>6<br>10	50% of 1 RM<br>30% of 1 RM<br>30% of 1 RM<br>50% of 1 RM<br>30% of 1 RM<br>50% of 1 RM<br>30% of 1 RM	NR	NR	3	3
Deluca etal., (2021)	progressive hamstring strengthening	NR				concentric advancing to eccentric		
Reilly et al., (2018)	progressive hamstring strengthening	NR	NR	NR	NR	eccentric	NR	NR
Sherry & Best et al., (2004)	Prone leg curls<br>Hip extension in standing with knee straight<br>Non–weight-bearing ''foot catches''	3	10	lerate level of resista	NR	concentric/ eccentric	3	8
Standert et al., (2012)	lunges<br>jumps	NR	NR	NR	NR	NR	3	3
Fredericson et al. (2005)	Double leg bridge<br>single leg bridge<br>isotonic open chain exercises	NR	NR	progressive	NR	isometric progressing to eccentric	NR	24
	Single leg romanian deadlift	3	6	progressive	NR	eccentric	4	16
	standing cable hip extension	3	6	progressive	NR	concentric	4	16
Silder et al., (2013)	Phase 1: increasing effort hamstring isometrics	10	10 second	BW	NA	isometric		6
	Phase 1: bilateral supine heel slides	1	15	BW	NA	concentric/ eccentric	5	6
	Phase 2: prone hamstring curls	3	12	moderate intensity	NR	concentric/ eccentric	5	6
	Phase 2: prone hip extension off bed	3	12	moderate intensity	NR	concentric/ eccentric	5	6
	Phase 2: prone leg lift and knee curl	3	12	moderate intensity	NR	concentric/ eccentric	5	6
	Phase 3: nordic with drop curl progression	2	8	high intensity	NR	eccentric	5	6
	Phase 3: Prone foot catches with ankle weight	2	10-20	high intensity	NR	eccentric	5	6
	Phase 3: prone hip extension off edge of bed	2	10-20	high intensity	NR	eccentric	5	6
	Phase 3: standing 1 foot catches	2	20	high intensity	NR	eccentric	5	6

Abbrevations: rating of pereived exertion (RPE), * performed twice daily, Repetitition Maximum (RM), Progressive Agility and Trunk Stabilization (PATS), Stretching and Strengthening (STS), Body Weight (BW)

Supplementary Table S6: *Lumbopelvic Stabilization Interventions*

LUMBOPELVIC STABILITY INTERVENTIONS					
**Author**	**Exercise**	**Sets**	**Repetitions**	**Hold Time (seconds)**	**Frequency / week**
Kreuger et al., (2020)	NA	NA	NA	NA	NA
Jayasleen et al.,(2014)	Swiss ball bridges<br>single leg romanian deadlift on half foam roll<br>standing 4-way hip on half foam roll + theraband<br>walking lunges	3	10 -15	NA	7
	side planks<br>planks<br>single leg stance	NR	NR	varied depending on whether proper form could be maintained	7
Mitchkash et al., (2020)	NR	NR	NR	NR	as tolerated
Cushman et al., (2015)	Phase 1 (four weeks): plank,<br>side plank<br>single arm plank<br>Swiss ball hamstring curl<br>single leg swiss ball curl	3-5	NA	30 - 60	7
McCormack et al., (2012)	NA	NA	NA	NA	NA
Cacchio et al., (2011)	NA	NA	NA	NA	NA
Deluca etal., (2021)	core/pelvic stabilization	NR	NR	NR	NR
Reilly et al., (2018)	NA	NA	NA	NA	NA
Sherry & Best et al., (2004)	Prone abdominal body bridge (performed by using abdominal and hip muscles to hold the body in a face-down straight-plank position with the elbows and feet as the only point of contact)<br>Single-leg stand progressing from eyes open to eyes closed<br>Supine extension bridge (performed by using abdominal and hip muscles to hold the body in a supine hook lying position with the head, upper back, arms, and feet as the points of contact)<br>Side bridge	4	NA	20	7
	Proprioceptive neuromuscular facilitation trunk pull-downs with Thera-Band<br>Push-up stabilization with trunk rotation (performed by starting at the top of a full push-up, then maintain this position with 1 hand while rotating the chest toward the side of the hand that is being lifted to point toward the ceiling, pause and return to the starting position)	2	15	NA	3
Standert et al., (2012)	NA	NA	NA	NA	NA
Fredericson et al. (2005)	swiss ball hamstring curl (double leg, single leg)<br>prone plank with hip extension	5	NA	10	NR
Askling et al., (2014)	single leg romanian deadlift	3	6	NA	4
Silder et al., (2013)	Phase 1: side plank	5	NA	10	5
	Phase 1: forarm plank	5	NA	10	5
	Phase 1: standing single leg balance	10	NA	5	5
	Phase 2: rotating body bridge	2	10	NA	5
	Phase 2: supine bent knee bridge walk outs			NA	5
	Phase 2:<br>lunge walk with trunk rotation and T lift	4	8	NA	5
	Phase 3: rotating body bridgge with dumbell	2	10	NA	5
	Phase 3: supine single leg chair bridge	3	15	NA	5
	Phase 3: single leg windmill touches with dumbell	4	8	NA	5
	Phase 3: lunge walk with trunk rotation and T lift	2	10	NA	5

Abbrevations: rating of pereived exertion (RPE), Not Applicable (NA), Not Reported (NR)

Supplementary Table S7: *Stretching Interventions*

STRETCHING INTERVENTIONS				
**Author**	**Stretch**	**Sets**	**Hold Time (seconds)**	**Frequency day / week**
Kreuger et al., (2020)	NA	NA	NA	NA
Jayasleen et al.,(2014)	NA	NA	NA	NA
Mitchkash et al., (2020)	NA	NA	NA	NA
Cushman et al., (2015)	hamstring stretch	1	180	7
McCormack et al., (2012)	supine hamstring stretch<br>contract relax hamstring stretch	2	30	7
Cacchio et al., (2011)	sitting hamstring stretch with anterior pelvic tilt<br>standing hamstring stretch with anterior pelvic tilt and side to side rotations<br>contract relax in standing with foot on stool	4	20	3
Deluca etal., (2021)	gastrocnemius/soleus stretching	NR	NR	1
Reilly et al., (2018)	NA	NA	NA	NA
Sherry & Best et al., (2004)	Supine hip flexion with knee extension stretch<br>Standing hip flexion with knee extension stretch with slow side-to-side rotation during the stretch<br>Contract-relax hamstring stretch in standing with foot on stool<br>Submaximal isometric hamstring sets, 10 reps for 10 sec held at 20° knee flexion and 60° knee flexion while lying<br>Phase 2: Supine hip flexion with knee extension stretch<br>Standing hip flexion with knee extension stretch with slow side to side rotation	4	20	3
Standert et al., (2012)	general stretching	NR	NR	NR
Fredericson et al. (2005)	NR	NR	NR	NR
Askling et al., (2014)	slow knee extension at 90 deg hip flexion	3 (12 reps)		7*
	hamstring contract/relax in standing with injured leg on high support surface	3 (4 reps)	10	7*
Silder et al., (2013)	NA	NA	NA	NA

Abbrevations: rating of pereived exertion (RPE), * performed twice a day, Not Applicable (NA), Not Reported (NR)

Supplementary Table S8: *Endurance/ Return To Run Progression*

ENDURANCE/ PLYOMETRIC INTERVENTIONS					
**Author**	**Endurance/ Agility Training**	**Return to Run Progression**	**Pace**	**Frequency**	**Intensity**
Kreuger et al., (2020)	NA	NA	NA	NA	NA
Jayasleen et al.,(2014)	NA	NA	NA	NA	NA
Mitchkash et al., (2020)	NA	NA	NA	NA	NA
Cushman et al., (2015)	Phase 1 (four weeks) : Swim cycle	Increasing mileage by 0.5 mi per day each week , starting at 0.5 mi distance	2 min slower than typical	every other day	painfree
McCormack et al., (2012)	NA	NA	NA	NA	NA
Cacchio et al., (2011)	NA	NA	NA	NA	NA
Deluca etal., (2021)	NA	NA	NA	NA	NA
Reilly et al., (2018)	running	as tolerated	NR	NR	NR
Sherry & Best et al., (2004)	sidestepping<br>grapevine stepping (lateral stepping with the trail leg going over the lead leg and then under the lead leg), both directions<br>steps forward and backward over a tape line while moving sideways<br>Phase 2* sidestepping<br>grapevine stepping<br>steps forward and backward while moving sideways	NA	NA	NA	Phase 1: low to moderate<br>Phase 2: moderate to high
Standert et al., (2012)	NA	NA	NA	NA	NA
Fredericson et al. (2005)	running	week 1: walk 5 min then run 1 mi x 5 bouts<br>week 2: if no pain, walk 5 and run 5 on alternate days working up to x 5 bouts<br>week 3: advance to 20 min jog<br>week 4: 20 min jog<br>week 4-8: gradual increase in running speed/ acceleration	week 1: 2 min per mile slower than typical<br>week 2: 1 min per mile slower than typical<br>week 3: 1 min per mile slower than typical<br>week 4: typical training pace	no more than 5 day / week	
Askling et al., (2014)	cycling	jogging 40 m x 10 with short strides<br>10 x 10 m forward / backward accellerations<br>once painfree progressing to<br>high speed running 6 x 20 m, 4 x 40 m, 2 x 60 m	NR	3 day / week	painfree
Silder et al., (2013)	Phase 1: 10 m back and forth side shuffle<br>10 m back and forth grape vine<br>fast foot in place<br>Phase 2: 10 back and forth side shuffle<br>10 m back and forth grapevine<br>10 m boxer shuffle<br>Phase 3: 30 m back and forth sideshuffle<br>30 m back and forth grapevine<br>10 m boxer shuffle<br>foward/ backward acclerations	Level 1 - 6: decreasing acceleration/ deceleration distance, starting at 40 m<br>Level 7-12: decreasing acceleration/ deceleration distance, starting at 40 m	Level 1-6: constant speed at 75% of max<br>Level 7-12: constant speed, 9% of max	3 day / week	Phase 1: low to moderate<br>Phase 2: moderate to high<br>Phase 3: moderate to high

Abbrevations: rating of pereived exertion (RPE), Not Applicable (NA), Not Reported (NR)

Supplementary Table S9: ***Modality Intervention***

MODALITY INTERVENTION							
Author	Modality	Shockwave Pressure (bars)	Shockwave Frequency (Hz)	Frequency of sessions / week	Number of Treatment Sessions	Location of Tx	Duration of Tx (min)
Kreuger et al., (2020)	NA	NA	NA	NA	NA	NA	NA
Jayasleen et al.,(2014)	TDN (0.3 x 50 mmm solid filament needle)	NA	NA	na	3	medial / lateral hamstrings adductor magnus	10 - 15
Mitchkash et al., (2020)	ESWT	2-5	12-15	1	3 - 6	proximal hamstring tendon	NR
Cushman et al., (2015)	NA	NA	NA	NA	NA	NA	NA
McCormack et al., (2012)	AYSTM	NA	NA	2	16	hamstring muscles in prone and proximal hamstring insertion	15-20
Cacchio et al., (2011)	ESWT	4	10	4	16	proximal hamstring tendon	NR
Deluca etal., (2021)	ESWT	2.5-5	15	1	4	proximal hamstring tendon	NR
Reilly et al., (2018)	RSWT	4.5	15	1	5	point of max tenderness	NR
Sherry & Best et al., (2004)	NA	NA	NA	NA	NA	NA	NA
Standert et al., (2012)	ESWT	2500 shocks per session at an energy flux density of 0.18 mJ/mm2	NA	NR	4	point of max tenderness	NR
Fredericson et al. (2005)	corticosteriod injection (	NA	NA	NA	1	distal to the ischial attachment	NA
Askling et al., (2014)	NA	NA	NA	NA	NA	NA	NA
Silder et al., (2013)	NA	NA	NA	NA	NA	NA	NA

Abbrevations: rating of pereived exertion (RPE), Trigger Point Dry Needling (TDN), Extracorporeal shockwave treatment (ESWT), Augmented Soft tissue Manipulation (AYSTM), Radial Shockwave Therapy (R-SWT), Combined Shockwave Therapy (C-SWT)

Supplementary Table S10: ***Pain Outcomes*.**

Author	Pain Scale	Treatment Group n=	Intragroup difference (change)	Stastical Significance	Clinical Signficance	Between group difference	Stastical Significance	Clinical Signficance	final VAS
Kreuger et al., (2020)	VAS	1	12 week: 6 pts	NR	Yes not stated however	NA	NA	NA	2 out of 10
Jayasleen et al.,(2014)	VAS	2	N1: 4 pts<br>N2: 6 pts	NR	N	NR	NR	NR	0/10
Mitchkash et al., (2020)	NA	32<br>responder n= 22<br>nonresponder n=10	NA	NA	NA	NA	NA	NA	NA
Cushman et al., (2015)	VAS	1	week 4: 0 pts<br>after 4 weeks of treadmill exercise (wk 8):7 pts<br>after 8 weeks of treadmill exercise (week 12): 7 pts	NR	NR	NR	NR	NR	0/10
McCormack et al., (2012)	VAS	1	6/10 pts	NR	NR	NR	NR	NR	0/10
Cacchio et al., (2011)	VAS (0-10)	n = 20 TCT<br>n=20 SWT	SWT: 7.1 +/- 1.1<br>TCT: 1.0 +/- 1.9	SWT: p=<.001<br>TCT: 0.76	Y for SWT group at 3 mo	p=<.001	Y	Y	SWT: 2.1<br>tct: 6.8
Cacchio et al., (2011)	NPRS (1-7)	n = 20 TCT<br>n=20 SWT	SWT: 5.1+/-0.8<br>TCT: 5.3 +/- 1.0	SWT: p=<.001<br>TCT: 0.87	Y for SWT group at 3 mo	p=<.001	Y	Y	SWT: 1.8<br>TCT: 5.5
Deluca etal., (2021)	NR	NR	NR	NR	NR	NR	NR	NR	NR
Reilly et al., (2018)	VAS	N=1	Pain expressed as present or absent; present on initial evaluation with running	NR	NR	NR	NR	NR	0/10
Sherry & Best et al., (2004)	NR	NR	NR	NR	NR	NR	NR	NR	NR
Standert et al., (2012)	NPRS (1-7)	n=30 control<br>n=20 SWT	SWT: 3.3<br>TCT: 0.2 worse	NR	NR	NR	NR	NR	SWT: 1.8<br>TCT: 5.5
Standert et al., (2012)	VAS (0-10)	n=30 control<br>n=20 SWT	SWT: 5 pt reductions<br>control: 0.2 reduction	NR	NR	NR	NR	NR	NR
Fredericson et al. (2005)	VAS	N=1	NR	NR	NR	NR	NR	NR	0/10
Askling et al., (2014)	peak palpation pain, distance to ischial tuberosity	n=28 in C protocol<br>n=28 in L protocol	NR	NR	NR	L protocol: 10 +/- 7 (9, 1 to 26)<br>C protocol: 10 +/- 7 (7, 2 to 24)	p=0.994(0.010)	p<0.05; Y	NR
Silder et al., (2013)	VAS	n=7 in PATS<br>n=4 in PRES	PATS: 9 pts<br>PRES: 5 pts	NR	NR	p=0.444	NR	NR	0/10

Supplementary Table S11: *VISA-H Outcome.*

Author	Outcome	Treatment Group n=	Intragroup difference	Stastical Significance	Clinical Signficance	Between group difference	Final VISA H
Kreuger et al., (2020)	VISAH	NR	NR	NR	NR	NR	NR
Jayasleen et al.,(2014)	VISAH	NR	NR	NR	NR	NR	NR
Mitchkash et al., (2020)	VISAH	32<br>responder n= 22<br>nonresponder n=10	26.1	NA	69% met MCID	NR	65.4/100
Cushman et al., (2015)	VISAH	1	60	NA	Y	NR	83/ 100
McCormack et al., (2012)	VISAH		NR	NR	NR	NR	NR
Cacchio et al., (2011)	VISAH	NR	NR	NR	NR	NR	NR
Deluca etal., (2021)	VISAH	RSWT: n=40<br>CSWT: n=23	RSWT: 31.76<br>CSWT: 34.7	RSWT: n=25 p<0.001<br>CSWT: n=13 p<0.001	Y<br>62.5% for RSWT<br>CSWT 56.5%	p = 0.641	RSWT: 71.6/100<br>CSWT: 71.07/100
Reilly et al., (2018)	VISAH	NR	NR	NR	NR	NR	NR
Sherry & Best et al., (2004)	NR	NR	NR	NR	NR	NR	NR
Standert et al., (2012)	NR	NR	NR	NR	NR	NR	NR
Fredericson et al. (2005)	NR	NR	NR	NR	NR	NR	NR
Askling et al., (2014)	NR	NR	NR	NR	NR	NR	NR
Silder et al., (2013)	NR	NR	NR	NR	NR	NR	NR

Supplementary Table S12: *Function Outcomes*.

Author	Outcome for Function	Treatment Group n=	Intragroup difference	Stastical Significance	Clinical Signficance	Between group difference	Stastical Significance	Final Outcome Score
Kreuger et al., (2020)	Sitting Tolerance	1	Initial Assessment: unable to to sit >30 min<br>12 weeks: 2/10 with sitting > 30 min<br>12 months: 2/10 with sitting > 60 min	NA	NA	NA	NA	NA
Jayasleen et al.,(2014)	LEFS	2	N1:13<br>N2: 11	Y	Y	NA	NA	N1: 80/80<br>N2:79/80
Mitchkash et al., (2020)	Sx Duration	responder n= 22<br>nonrespondder n=10	responder: 12.4 +/- 12.9<br>nonresponder: 25.4 +/- 36.8	NR	NR	NR	NR	NR
Cushman et al., (2015)	Sitting Tolerance	1	week 4: present<br>after 4 weeks of treadmill exercise (wk 8): absent<br>after 8 weeks of treadmill exercise (week 12): absent	NR	NR	NR	NR	NR
McCormack et al., (2012)	Jog Tolerance<br>LEFS	1	8 week: 2.5 mile without pain<br>week 12: 1 mile jog without pain<br>week 16: 2.5 mi run without pain<br>LEFS: 10	NR	Y	NR	NR	74/80
Cacchio et al., (2011)	Return to Sport	n = 20 TCT<br>n=20 SW	SWT: 80% return to preinject progressional level<br>TCT: none	NR	NR	NR	P <0.001	NR
Deluca etal., (2021)	NR	NR	NR	NR	NR	NR	NR	NR
Reilly et al., (2018)	Return to sport	n=1	Run ability: 12-15 km<br>four months: 100 km	NR	NR	NR	NR	back painfree at 10 months
Sherry & Best et al., (2004)	Reinjury rate	STST n=11<br>2 week: 6 (54.5%)<br>1 year: 7 (70%)<br>PATS n =13<br>2 week: 0%<br>1 yr: 1 (7.7%)	PATS: P= 0.00343 2 weeks<br>1 yr: p=0.0059	NR	NR	p=0.789	N	STST n=11<br>2 week: 6 (54.5%)<br>1 year: 7 (70%)<br>PATS n =13<br>2 week: 0%<br>1 yr: 1 (7.7%)
	Return to Sport (days)	NR	STST: 37.4 D<br>pats: 22.2 D	NR	NR	p=.245	N	"STST: 37.4 D<br>pats: 22.2 D "
Standert et al., (2012)	return to preinjury level	n= 30 control<br>n=20 SWT	Control: none<br>SWT: 80%	NR	NR	NR	NR	NR
Fredericson et al. (2005)	return to preinjury level	n=1	NR	NR	NR	NR	NR	6 month
Askling et al., (2014)	Return to sport (days)	n=16 in C protocol<br>n=16 in L protocol	L protocol: median: 62 days<br>C protocol: median: 120 days	NR	NR	<p. 01	N	86 days
Silder et al., (2013)	Return to Sport (days)	n=16 in PATS<br>n=13 in PRES	PRES: 28.8 days<br>PATS: 25.2 days	NR	NR	p=.512	N	NR
	Cross sectional injured area	n=16 in PATS<br>n=13 in PRES	PATS: 25.8% reduction<br>PRES: 28.6% reduction	PATS: p=-0.75; 95% CI: -1.2,-0.31)<br>PRES: p=-0.75, 95% CI: -0.98, -0.01)	N	p=0.438	N	No subject showed complete injury resolution with T2 MRI

Supplementary Table S13: ***Modality Intervention.***

MODALITY INTERVENTION							
Author	Modality	Shockwave Pressure (bars)	Shockwave Frequency (Hz)	Frequency of sessions / week	Number of Treatment Sessions	Location of Tx	Duration of Tx (min)
Kreuger et al., (2020)	NA	NA	NA	NA	NA	NA	NA
Jayasleen et al.,(2014)	TDN (0.3 x 50 mmm solid filament needle)	NA	NA	na	3	medial / lateral hamstrings adductor magnus	10 - 15
Mitchkash et al., (2020)	ESWT	2-5	12-15	1	3 - 6	proximal hamstring tendon	NR
Cushman et al., (2015)	NA	NA	NA	NA	NA	NA	NA
McCormack et al., (2012)	AYSTM	NA	NA	2	16	hamstring muscles in prone and proximal hamstring insertion	15-20
Cacchio et al., (2011)	ESWT	4	10	4	16	proximal hamstring tendon	NR
Deluca etal., (2021)	ESWT	2.5-5	15	1	4	proximal hamstring tendon	NR
Reilly et al., (2018)	RSWT	4.5	15	1	5	point of max tenderness	NR
Sherry & Best et al., (2004)	NA	NA	NA	NA	NA	NA	NA
Standert et al., (2012)	ESWT	2500 shocks per session at an energy flux density of 0.18 mJ/mm2	NA	NR	4	point of max tenderness	NR
Fredericson et al. (2005)	corticosteriod injection (	NA	NA	NA	1	distal to the ischial attachment	NA
Askling et al., (2014)	NA	NA	NA	NA	NA	NA	NA
Silder et al., (2013)	NA	NA	NA	NA	NA	NA	NA

Abbrevations: rating of pereived exertion (RPE), Trigger Point Dry Needling (TDN), Extracorporeal shockwave treatment (ESWT), Augmented Soft tissue Manipulation (AYSTM), Radial Shockwave Therapy (R-SWT), Combined Shockwave Therapy (C-SWT)

Supplementary Table S14: ***Pain Outcomes*.**

Author	Pain Scale	Treatment Group n=	Intragroup difference (change)	Stastical Significance	Clinical Signficance	Between group difference	Stastical Significance	Clinical Signficance	final VAS
Kreuger et al., (2020)	VAS	1	12 week: 6 pts	NR	Yes not stated however	NA	NA	NA	2 out of 10
Jayasleen et al.,(2014)	VAS	2	N1: 4 pts<br>N2: 6 pts	NR	N	NR	NR	NR	0/10
Mitchkash et al., (2020)	NA	32<br>responder n= 22<br>nonresponder n=10	NA	NA	NA	NA	NA	NA	NA
Cushman et al., (2015)	VAS	1	week 4: 0 pts<br>after 4 weeks of treadmill exercise (wk 8):7 pts<br>after 8 weeks of treadmill exercise (week 12): 7 pts	NR	NR	NR	NR	NR	0/10
McCormack et al., (2012)	VAS	1	6/10 pts	NR	NR	NR	NR	NR	0/10
Cacchio et al., (2011)	VAS (0-10)	n = 20 TCT<br>n=20 SWT	SWT: 7.1 +/- 1.1<br>TCT: 1.0 +/- 1.9	SWT: p=<.001<br>TCT: 0.76	Y for SWT group at 3 mo	p= <.001	Y	Y	SWT: 2.1<br>tct: 6.8
Cacchio et al., (2011)	NPRS (1-7)	n = 20 TCT<br>n=20 SWT	SWT: 5.1+/-0.8<br>TCT: 5.3 +/- 1.0	SWT: p=<.001<br>TCT: 0.87	Y for SWT group at 3 mo	p= <.001	Y	Y	SWT: 1.8<br>TCT: 5.5
Deluca etal., (2021)	NR	NR	NR	NR	NR	NR	NR	NR	NR
Reilly et al., (2018)	VAS	N=1	Pain expressed as present or absent; present on initial evaluation with running	NR	NR	NR	NR	NR	0/10
Sherry & Best et al., (2004)	NR	NR	NR	NR	NR	NR	NR	NR	NR
Standert et al., (2012)	NPRS (1-7)	n=30 control<br>n=20 SWT	SWT: 3.3<br>TCT: 0.2 worse	NR	NR	NR	NR	NR	SWT: 1.8<br>TCT: 5.5
Standert et al., (2012)	VAS (0-10)	n=30 control<br>n=20 SWT	SWT: 5 pt reductions<br>control: 0.2 reduction	NR	NR	NR	NR	NR	NR
Fredericson et al. (2005)	VAS	N=1	NR	NR	NR	NR	NR	NR	0/10
Askling et al., (2014)	peak palpation pain, distance to ischial tuberosity	n=28 in C protocol<br>n=28 in L protocol	NR	NR	NR	L protocol: 10 +/- 7 (9, 1 to 26)<br>C protocol: 10 +/- 7 (7, 2 to 24)	p=0.994(0.010)	p<0.05; Y	NR
Silder et al., (2013)	VAS	n=7 in PATS<br>n=4 in PRES	PATS: 9 pts<br>PRES: 5 pts	NR	NR	p=0.444	NR	NR	0/10

Supplementary Table S15: *VISA-H Outcome.*

Author	Outcome	Treatment Group n=	Intragroup difference	Stastical Significance	Clinical Signficance	Between group difference	Final VISA H
Kreuger et al., (2020)	VISAH	NR	NR	NR	NR	NR	NR
Jayasleen et al.,(2014)	VISAH	NR	NR	NR	NR	NR	NR
Mitchkash et al., (2020)	VISAH	32<br>responder n= 22<br>nonresponder n=10	26.1	NA	69% met MCID	NR	65.4/100
Cushman et al., (2015)	VISAH	1	60	NA	Y	NR	83/ 100
McCormack et al., (2012)	VISAH		NR	NR	NR	NR	NR
Cacchio et al., (2011)	VISAH	NR	NR	NR	NR	NR	NR
Deluca etal., (2021)	VISAH	RSWT: n=40<br>CSWT: n=23	RSWT: 31.76<br>CSWT: 34.7	RSWT: n=25 p<0.001<br>CSWT: n=13 p<0.001	Y<br>62.5% for RSWT<br>CSWT 56.5%	p = 0.641	RSWT: 71.6/100<br>CSWT: 71.07/100
Reilly et al., (2018)	VISAH	NR	NR	NR	NR	NR	NR
Sherry & Best et al., (2004)	NR	NR	NR	NR	NR	NR	NR
Standert et al., (2012)	NR	NR	NR	NR	NR	NR	NR
Fredericson et al. (2005)	NR	NR	NR	NR	NR	NR	NR
Askling et al., (2014)	NR	NR	NR	NR	NR	NR	NR
Silder et al., (2013)	NR	NR	NR	NR	NR	NR	NR

Supplementary Table S16: *Function Outcomes*.

Author	Outcome for Function	Treatment Group n=	Intragroup difference	Stastical Significance	Clinical Signficance	Between group difference	Stastical Significance	Final Outcome Score
Kreuger et al., (2020)	Sitting Tolerance	1	Initial Assessment: unable to to sit >30 min<br>12 weeks: 2/10 with sitting > 30 min<br>12 months: 2/10 with sitting > 60 min	NA	NA	NA	NA	NA
Jayasleen et al.,(2014)	LEFS	2	N1:13<br>N2: 11	Y	Y	NA	NA	N1: 80/80<br>N2:79/80
Mitchkash et al., (2020)	Sx Duration	responder n= 22<br>nonrespondder n=10	responder: 12.4 +/- 12.9<br>nonresponder: 25.4 +/- 36.8	NR	NR	NR	NR	NR
Cushman et al., (2015)	Sitting Tolerance	1	week 4: present<br>after 4 weeks of treadmill exercise (wk 8): absent<br>after 8 weeks of treadmill exercise (week 12): absent	NR	NR	NR	NR	NR
McCormack et al., (2012)	Jog Tolerance<br>LEFS	1	8 week: 2.5 mile without pain<br>week 12: 1 mile jog without pain<br>week 16: 2.5 mi run without pain<br>LEFS: 10	NR	Y	NR	NR	74/80
Cacchio et al., (2011)	Return to Sport	n = 20 TCT<br>n=20 SW	SWT: 80% return to preinject progressional level<br>TCT: none	NR	NR	NR	P <0.001	NR
Deluca etal., (2021)	NR	NR	NR	NR	NR	NR	NR	NR
Reilly et al., (2018)	Return to sport	n=1	Run ability: 12-15 km<br>four months: 100 km	NR	NR	NR	NR	back painfree at 10 months
Sherry & Best et al., (2004)	Reinjury rate	STST n=11<br>2 week: 6 (54.5%)<br>1 year: 7 (70%)<br>PATS n =13<br>2 week: 0%<br>1 yr: 1 (7.7%)	PATS: P= 0.00343 2 weeks<br>1 yr: p=0.0059	NR	NR	p=0.789	N	STST n=11<br>2 week: 6 (54.5%)<br>1 year: 7 (70%)<br>PATS n =13<br>2 week: 0%<br>1 yr: 1 (7.7%)
	Return to Sport (days)	NR	STST: 37.4 D<br>pats: 22.2 D	NR	NR	p=.245	N	"STST: 37.4 D<br>pats: 22.2 D "
Standert et al., (2012)	return to preinjury level	n= 30 control<br>n=20 SWT	Control: none<br>SWT: 80%	NR	NR	NR	NR	NR
Fredericson et al. (2005)	return to preinjury level	n=1	NR	NR	NR	NR	NR	6 month
Askling et al., (2014)	Return to sport (days)	n=16 in C protocol<br>n=16 in L protocol	L protocol: median: 62 days<br>C protocol: median: 120 days	NR	NR	<p. 01	N	86 days
Silder et al., (2013)	Return to Sport (days)	n=16 in PATS<br>n=13 in PRES	PRES: 28.8 days<br>PATS: 25.2 days	NR	NR	p=.512	N	NR
	Cross sectional injured area	n=16 in PATS<br>n=13 in PRES	PATS: 25.8% reduction<br>PRES: 28.6% reduction	PATS: p=-0.75; 95% CI: -1.2,-0.31)<br>PRES: p=-0.75, 95% CI: -0.98, -0.01)	N	p=0.438	N	No subject showed complete injury resolution with T2 MRI

Supplementary Table S17: ***Oxford Center for Evidenced-Based Medicine 2011 Level of Evidence.***

Question	Step 1 (Level 1*)	Step 2 (Level 2*)	Step 3 (Level 3*)	Step 4 (Level 4*)	Step 5 (Level 5)
**How common is the problem?**	Local and current random sample surveys (or censuses)	Systematic review of surveys that allow matching to local circumstances**	Local non-random sample**	Case-series**	n/a
**Is this diagnostic or monitoring test accurate?** (Diagnosis)	Systematic review of cross sectional studies with consistently applied reference standard and blinding	Individual cross sectional studies with consistently applied reference standard and blinding	Non-consecutive studies, or studies without consistently applied reference standards**	Case-control studies, or "poor or non-independent reference standard**	Mechanism-based reasoning
**What will happen if we do not add a therapy?** (Prognosis)	Systematic review of inception cohort studies	Inception cohort studies	Cohort study or control arm of randomized trial*	Case-series or case-control studies, or poor quality prognostic cohort study**	n/a
**Does this intervention help?** (Treatment Benefits)	Systematic review of randomized trials or *n*-of-1 trials	Randomized trial or observational study with dramatic effect	Non-randomized controlled cohort/follow-up study**	Case-series, case-control studies, or historically controlled studies**	Mechanism-based reasoning
**What are the COMMON harms?** (Treatment Harms)	Systematic review of randomized trials, systematic review of nested case-control studies, *n*-of-1 trial with the patient you are raising the question about, or observational study with dramatic effect	Individual randomized trial or (exceptionally) observational study with dramatic effect	Non-randomized controlled cohort/follow-up study (post-marketing surveillance) provided there are sufficient numbers to rule out a common harm. (For long-term harms the duration of follow-up must be sufficient.)**	Case-series, case-control, or historically controlled studies**	Mechanism-based reasoning
**What are the RARE harms?** (Treatment Harms)	Systematic review of randomized trials or *n*-of-1 trial	Randomized trial or (exceptionally) observational study with dramatic effect			
**Is this (early detection) test worthwhile?** (Screening)	Systematic review of randomized trials	Randomized trial	Non -randomized controlled cohort/follow-up study**	Case-series, case-control, or historically controlled studies**	Mechanism-based reasoning

*** Level may be graded down on the basis of study quality, imprecision, indirectness (study PICO does not match questions PICO), because of inconsistency between studies, or because the absolute effect size is very small; Level may be graded up if there is a large or very large effect size.**

**** As always, a systematic review is generally better than an individual study.**

How to cite the Levels of Evidence Table

OCEBM Levels of Evidence Working Group*. "The Oxford 2011 Levels of Evidence".

Oxford Centre for Evidence-Based Medicine. http://www.cebm.net/index.aspx?o=5653

** OCEBM Table of Evidence Working Group = Jeremy Howick, Iain Chalmers (James Lind Library), Paul Glasziou, Trish Greenhalgh, Carl Heneghan, Alessandro Liberati, Ivan Moschetti, Bob Phillips, Hazel Thornton, Olive Goddard and Mary Hodgkinson*

Supplementary Table S18: *Grades of Recommendation.*

Grades of Recommendation		Strength of Evidence	Level of Obligation
A	Strong evidence	A preponderance of level I and/or level II studies support the recommendation. This must include at least 1 level I study	Must or should
B	Moderate evidence	A single high-quality randomized controlled trial or a preponderance of level II studies support the recommendation	Should
C	Weak evidence	A single level II study or a preponderance of level III and IV studies, including statements of consensus by content experts, support the recommendation	May
D	Conflicting evidence	Higher-quality studies conducted on this topic disagree with respect to their conclusions. The recommendation is based on these conflicting study results	
E	Theoretical/foundational evidence	A preponderance of evidence from animal or cadaver studies, from conceptual models/principles, or from basic sciences/bench research support this conclusion	May
F	Expert	opinion Best practice based on the clinical experience of the guidelines development team supports this conclusion	May
